# Sox2 promotes expression of the ST6Gal-I glycosyltransferase in ovarian cancer cells

**DOI:** 10.1186/s13048-019-0574-5

**Published:** 2019-10-14

**Authors:** Kaitlyn A. Dorsett, Robert B. Jones, Katherine E. Ankenbauer, Anita B. Hjelmeland, Susan L. Bellis

**Affiliations:** Department of Cell, Developmental and Integrative Biology, University of Alabama at Birmingham, MCLM 350, 1918 University Boulevard, Birmingham, AL 35294 USA

**Keywords:** Sialic acid, ST6Gal-I, Sox2, 3q26 amplicon, Cancer stem cells, Ovarian cancer

## Abstract

**Background:**

The ST6Gal-I glycosyltransferase, which adds α2–6-linked sialic acids to *N*-glycosylated proteins is upregulated in a wide range of malignancies including ovarian cancer. Prior studies have shown that ST6Gal-I-mediated sialylation of select surface receptors remodels intracellular signaling to impart cancer stem cell (CSC) characteristics. However, the mechanisms that contribute to ST6Gal-I expression in stem-like cancer cells are poorly understood.

**Results:**

Herein, we identify the master stem cell transcription factor, Sox2, as a novel regulator of ST6Gal-I expression. Interestingly, *SOX2* and *ST6GAL1* are located within the same tumor-associated amplicon, 3q26, and these two genes exhibit coordinate gains in copy number across multiple cancers including ~ 25% of ovarian serious adenocarcinomas. In conjunction with genetic co-amplification, our studies suggest that Sox2 directly binds the *ST6GAL1* promoter to drive transcription. ST6Gal-I expression is directed by at least four distinct promoters, and we identified the P3 promoter as the predominant promoter utilized by ovarian cancer cells. Chromatin Immunoprecipitation (ChIP) assays revealed that Sox2 binds regions proximal to the P3 promoter. To confirm that Sox2 regulates ST6Gal-I expression, Sox2 was either overexpressed or knocked-down in various ovarian cancer cell lines. Sox2 overexpression induced an increase in ST6Gal-I mRNA and protein, as well as surface α2–6 sialylation, whereas Sox2 knock-down suppressed levels of ST6Gal-I mRNA, protein and surface α2–6 sialylation.

**Conclusions:**

These data suggest a process whereby *SOX2* and *ST6GAL1* are coordinately amplified in cancer cells, with the Sox2 protein then binding the *ST6GAL1* promoter to further augment ST6Gal-I expression. Our collective results provide new insight into mechanisms that upregulate ST6Gal-I expression in ovarian cancer cells, and also point to the possibility that some of the CSC characteristics commonly attributed to Sox2 may, in part, be mediated through the sialyltransferase activity of ST6Gal-I.

## Introduction

Ovarian cancer is the deadliest gynecological malignancy due primarily to late detection and development of drug resistance [1, 2]. While most ovarian cancer patients respond initially to treatment, acquired resistance is common, leading to tumor recurrence [2]. Cancer stem cells (CSCs) are thought to be principal drivers of ovarian cancer progression and recurrence due to their high degree of resistance to both chemotherapy and microenvironmental stressors such as hypoxia [3, 4]. Thus, extensive research is focused on the molecular mechanisms that promote a CSC phenotype. However, the potential role of glycosyltransferases, and their cognate glycan structures, in CSC behavior has received limited attention.

One of the predominant glycosyltransferases upregulated in ovarian and other cancers is ST6Gal-I, a sialyltransferase that adds α2–6-linked sialic acids to *N*-glycosylated proteins destined for the cell surface or secretion [5–8]. High expression of ST6Gal-I is correlated with decreased overall and progression free survival in patients with high-grade serous ovarian carcinoma [9, 10], the most common and aggressive subtype [1]. ST6Gal-I regulates tumor cell phenotype by modulating the sialylation, and therefore function, of key receptors that drive malignant cell behaviors [11–17]. We and others have shown that ST6Gal-I activity confers all of the hallmark features of a CSC including increased expression of canonical CSC markers [18], invasive potential [16, 19], tumor-initiating potential [9, 20], and resistance to hypoxia, chemotherapeutics, and radiation [14, 20–25].

In addition to CSCs, ST6Gal-I expression is enriched in select stem/progenitor niches within normal tissues, such as the basal layer of epidermis and fallopian tube fimbriae [9, 18]. In contrast, ST6Gal-I expression is negligible in many differentiated cell populations such as ovarian surface epithelium and pancreatic acinar cells [9]. Despite these cell type-specific differences in the levels of ST6Gal-I, there is limited knowledge regarding the mechanisms that regulate ST6Gal-I expression. ST6Gal-I regulation is known to be complex, as there are multiple ST6Gal-I mRNA isoforms [26–29]. These diverse mRNA species are transcribed from at least four promoters: P1, which is liver selective; P2, which is utilized exclusively by B cells; P3, a ubiquitously-utilized promoter; and P4, which is active in the mammary gland during lactation [26–32]. In cancer cells, ST6Gal-I upregulation appears to be directed primarily by the P1 or P3 promoter [30, 33–35].

ST6Gal-I is dramatically upregulated upon transduction of somatic cells with the four Yamanaka factors to generate induced pluripotent stem cells (iPSCs) [18, 36], and knock-down of ST6Gal-I hinders transition to pluripotency [37]. One of the Yamanaka factors is Sox2, suggesting potential regulation of ST6Gal-I by this stem cell-associated transcription factor. Moreover, several studies have reported a strong correlation between the mRNA levels of Sox2 and ST6Gal-I [38–40]. Like ST6Gal-I, Sox2 is upregulated in ovarian cancer [41–43], and its expression is particularly enriched in the CSC population of many different malignancies [44–46]. Both Sox2 and ST6Gal-I play causal roles in promoting CSC characteristics [9, 25, 47]. Interestingly, the *SOX2* and *ST6GAL1* genes lie within the same amplicon, referred to as “3q26”, which spans from 3q26-3q29 [48–50]. The 3q26 amplicon is one of the most commonly amplified genomic regions across many cancer types, and it functions as a multigenic driver of human cancer [48]. Amplification of the 3q26 region represents an early event in tumorigenesis, and has been associated with enhanced aggressiveness and stem-like properties of epithelial cancers [48, 51]. While several genes within this amplicon have been implicated in neoplastic transformation, such as *SOX2*, *PI3KCA* and *ECT2* [48], the potential role of ST6Gal-I in the tumor-promoting activity of the 3q26 amplicon has gone unnoticed.

In the current study we investigated a novel function for Sox2 in regulating the expression of ST6Gal-I. We first analyzed The Cancer Genome Atlas (TCGA) databases for copy number alterations in *SOX2* and *ST6GAL1* and showed that these two genes are coordinately amplified in patient specimens across a wide range of cancer types, including ovarian cancer. Furthermore, protein levels of Sox2 and ST6Gal-I were found to strongly correlate in established ovarian cancer cell lines. We next interrogated a possible direct interaction between Sox2 and ST6Gal-I by performing Chromatin Immunoprecipitation (ChIP) assays, which revealed that Sox2 binds to sequences proximal to the *ST6GAL1* P3 promoter. To confirm that Sox2 regulates ST6Gal-I expression, Sox2 was knocked-down in Pa-1 ovarian cancer cells, which have high endogenous ST6Gal-I, or overexpressed in Skov3 ovarian cancer cells, which have relatively low ST6Gal-I expression. Sox2 knock-down reduced ST6Gal-I mRNA and protein expression, and correspondingly diminished surface α2–6 sialylation, whereas Sox2 overexpression increased ST6Gal-I mRNA and protein, and enhanced surface sialylation. These data suggest that Sox2 is a key transcription factor responsible for upregulating ST6Gal-I expression in ovarian cancer cells.

## Materials and methods

### Cell culture

Skov-3, Pa-1, OVCAR3, OVCAR4, and OVCAR5 cell lines were obtained from ATCC. A2780 parental cells (IP2) and cisplatin resistant cells (CP20) were generously donated by Dr. Charles Landen (University of Virginia). Cells were grown in RPMI (Skov-3, A2780, OVCAR4) or DMEM (Pa-1, OVCAR5) media containing 10% fetal bovine serum (FBS, Atlanta Biologicals) and antibiotic/antimycotic supplements (Invitrogen). OVCAR3 cells were grown in RPMI with 20% FBS and 0.01 mg/mL of bovine insulin (Sigma). Normal human astrocytes (NHA, Lonza) were cultured in AGM media, and immortalized neural progenitor cells (NPC, Millipore) were propagated in DMEM/F12 supplemented with EGF, FGF and Gem21 (Gemini Bio-Products). Stable polyclonal cell lines with either forced expression of Sox2 (GeneCopoeia), or shRNA against Sox2 (Sigma), were created by lentiviral transduction followed by puromycin selection. Cells with inducible Sox2 expression were generated using lentivirus harboring a tetracycline-inducible Sox2 construct (GeneCopoeia) followed by selection with blasticidin. Sox2 expression was induced in this latter cell line with 1 μg/ml doxycycline. In a pilot experiment, dox-induced Sox2 expression was measured at multiple time points, and based on these data, all further dox treatments were conducted at 96 h. Modulation of Sox2 expression in these various cell models was confirmed by immunoblotting.

### Immunoblotting

Cells were lysed in RIPA buffer (Thermo Fisher Scientific) containing protease and phosphatase inhibitors (Sigma). Protein quantification was performed by BCA assay (Pierce). Lysates were resolved by SDS-PAGE and transferred to polyvinylidene difluoride (PVDF) membrane (Sigma). Blots were blocked in 5% non-fat dried milk and then incubated with primary antibodies against Sox2 (Cell Signaling, 3579S), ST6Gal-I (R&D Systems, goat polyclonal, AF5924), or β-tubulin (AbCam). Blots were subsequently incubated with the appropriate secondary antibody (Cell Signaling, anti-rabbit; R&D systems, anti-goat), and then developed using either Clarity (Bio-Rad) or SuperSignal West Femto (Pierce) enhanced chemiluminescence (ECL) substrates.

### mRNA isoform analysis

RNA was isolated from cells using the Ambion RNA extraction kit (Life Technologies). cDNA was then synthesized according to the vendor protocol (Promega), and PCR was used to amplify specific ST6Gal-I isoforms. Primers with the following sequences were obtained from Integrated DNA Technologies:

- H isoform: forward: GTCTCTTATTTTTTGCCTTTGCAG, reverse: CCACACACAGATGACTGCAA
- YZ isoform: forward: AGTCCAGGGAGAAGTGGTGA, reverse: CCACACACAGATGACTGCAA
- X isoform: forward: CTTCTCCCATACCTTGCTCTACA, reverse: GAAGATGTGTTCAGGGAAGTCAC
- Coding region: forward: TATCGTAAGCTGCACCCCAATC, reverse: TTAGCAGTGAATGGTCCGGAAG.
- GAPDH: forward: TGGTATCGTGGAAGGACTCA; reverse: AGTGGGTGTCGCTGTTGAAG.

Isoform-specific PCR products were visualized on a 1.2% agarose gel with ethidium bromide.

### qRT-PCR

For analyses of ST6Gal-I and Sox2 mRNA expression, TaqMan Fast Master Mix (Thermo Fisher Scientific) was used and primers were purchased from Applied Biosystems: ST6Gal-I (Assay ID: Hs00949382_m1) and Sox2 (AssayID: Hs00602736_m1); mRNA expression was normalized to GAPDH (Assay ID: HS02786624_g1). At least 3 independent experiments were conducted, with each individual experiment performed with 3 technical replicates. Statistical significance was defined as *p* < 0.05 based on a Student’s T Test.

### Chromatin Immunoprecipitation (ChIP)

Cells were plated at a density of 1 × 10^8^ cells per 150 cm^2^ dish, and allowed to adhere overnight. Cells were fixed with 3.7% paraformaldehyde (Thermo Fisher Scientific) for 10 min, and then fixation was stopped by adding 2.5 M glycine. Cells were washed with phosphate-buffered saline (PBS) containing protease inhibitors, removed from the plate using a cell scraper, and centrifuged at 1000 x g for 5 min. Cell pellets were then resuspended in hypotonic buffer (10 mM HEPES, pH 7.9; 1.5 mM MgCl_2_, 10 mM KCl, 2.5 M glycine) with fresh protease inhibitors, and incubated on ice for 5 min. NP-40 (Sigma Aldrich) was added to the solution at a final concentration of 0.5%. Samples were incubated on ice for 5 min. Cells were centrifuged and the pellets resuspended in TE buffer (Thermo Fisher Scientific) with fresh protease inhibitors. Samples were then sonicated using a BioRupter for 20 cycles of 30 s on; 30 s off; with temperature remaining at 4 °C. Following sonication, samples were centrifuged at 4 °C for 15 min and cell pellet debris was removed. 5% of the sample was removed for the input control. A 1:1 volume of 2x RIPA buffer was then added to each sample along with either a ChIP-validated anti-Sox2 antibody (Cell Signaling, 5024S) or a nonspecific IgG control antibody (Cell Signaling, 3900S). Chromatin samples were incubated with the antibodies for 2 h at 4 °C with rotation. Samples were then incubated with Protein G dynabeads (Invitrogen) overnight at 4 °C with rotation. The antibody/chromatin complexes were collected by placing the samples in a magnet (DynaMag, Life Technologies), and complexes were washed with the following series of buffers at 4 °C with rotation for 5 min each: Low salt buffer (0.1% SDS, 1% Triton-X-100, 1% sodium deoxycholate, 1 mM EDTA, 10 mM Tris HCl, pH 8.1); High salt buffer (0.1% SDS, 1% Triton-X-100, 2 mM EDTA, 20 mM Tris HCl, pH 8.1, 150 mM NaCl), and LiCl Buffer (0.25 M LiCl, 1% NP-40, 1% sodium deoxycholate, 1 M EDTA, 10 mM Tris HCl, pH 8.1). Complexes were subsequently washed twice with TE buffer. Following the washes, samples were incubated with fresh elution buffer (10% SDS, 1 mM NaHCO_3_) at room temperature for 15 min; this step was repeated once. Eluates were collected, and crosslinking reversed by adding 0.2 M NaCl and 10 mg/mL RNase A (Thermo Fisher Scientific) and incubating at 65 °C for 4 h. Protein was then degraded by adding proteinase K (20 mg/mL) to a final concentration of 50 μg/ml at 60 °C for 1 h. DNA was purified using the SimpleChIP DNA purification kit (Cell Signaling) according to the manufacturer’s instructions. PCR was conducted using the SYBR®Green system (Thermo Fisher Scientific). Primer sequences for PCR are as follows:

- 1A: forward: TTGTGGCTGTGATCCTTTCA, reverse: CTGCACAGATGGGCTGATAA;
- 1B: forward: TGCCCCCACTCTGCTTTATC, reverse: TTTAAGCACACAGGGATGGCT;
- 2A: forward: GGTTACTCCAGGCTGAGTCG, reverse: CTCCAGGAGGTGAAGGTGAG;
- 2B: forward: CCGCTTGGGCATCAGACTAA, reverse: CGACTCAGCCTGGAGTAACC;
- 3A: forward: GAGGAGGGATTGGGTTTGTT, reverse: TCCTTAAACAGCACAGTTCCA;
- 3B: forward: ACTGTGCTGTTTAAGGATCAACTG, reverse: AACTGGGCTCCACAATGCAA.

Data were normalized to the percent input, and then values for the nonspecific IgG ChIP were normalized to 1.0.

### Flow Cytometry

Cells were detached from tissue culture plates with Accutase (Corning) and washed with cold PBS. Cells were blocked in 1% bovine serum albumin (BSA) in PBS for 30 min on ice. Cells were stained with Dylight 649-conjugated SNA (EY labs), a lectin that specifically binds α2–6 linked sialic acids. Cells were stained with SNA-649 at dilutions of 1:200 or 1:400, depending on cell type, in 0.1% BSA in PBS for 40 min on ice. Binding of SNA-649 was quantified using the LSRII flow cytometer courtesy of the UAB Flow Cytometry Core.

### TCGA analyses

The presence of copy number alterations for *ST6GAL1* and *SOX2* in human tumor tissues was evaluated in TCGA datasets using cBioportal [52, 53]. cBioportal was used to obtain a GISTIC score for these two genes across 74 cancer cohorts (the total number of datasets for which copy number data were available). A GISTIC score of + 1 or + 2 was considered to indicate an amplification (colored red), and a score of − 1 or − 2 indicates a deletion (colored blue). Percent of alteration was calculated from the percentage of samples in each cohort that contained an amplification or deletion.

## Results

### SOX2 and ST6GAL1 are co-amplified in many human cancers including ovarian cancer

**Fig. 1:**
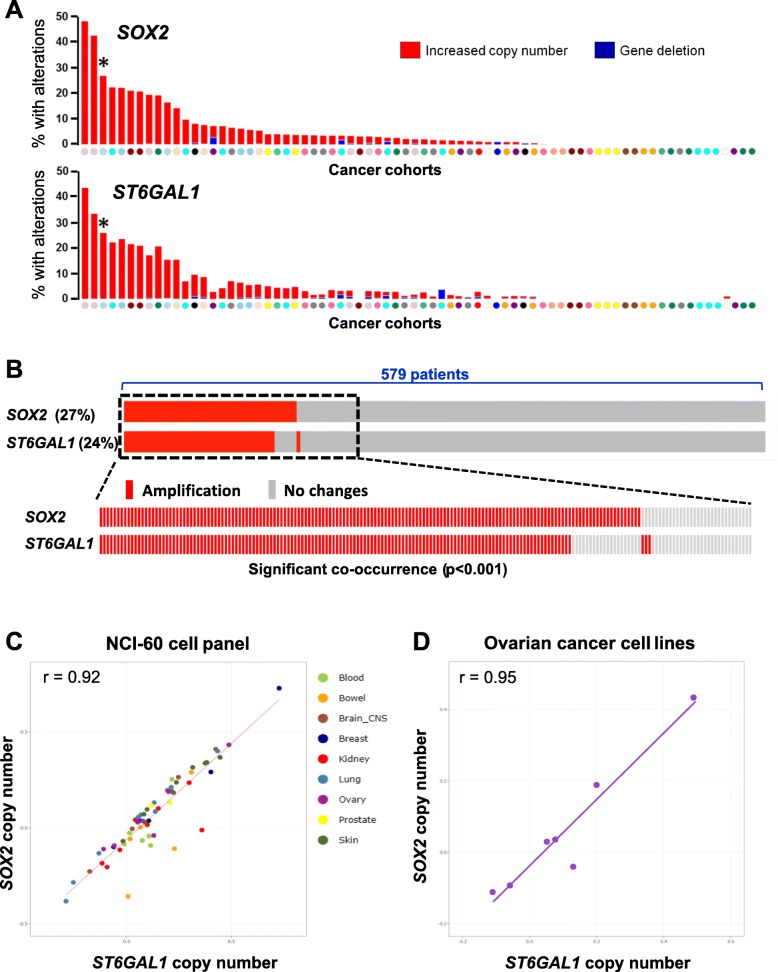
*SOX2* and *ST6GAL1* co-amplification. **a** Aligned TCGA datasets showing copy number alterations for *SOX2* (upper panel) and *ST6GAL1* (lower panel). Colored circles represent the various types of cancer cohorts. The cohorts were ordered based on the frequency *SOX2* amplification in the distinct cohorts and the same cohort order was then used to graph the data for *ST6GAL1* copy number variations. Gene amplification is shown in red, deletion in blue. An ovarian serous carcinoma cohort is marked by *. **b** Aligned individual patient tumors from the ovarian serous carcinoma cohort (*) showing *SOX2* and *ST6GAL1* co-amplification and co-occurrence. **c** *SOX2* and *ST6Gal1* copy number in the NCI-60 series of established cancer cell lines. **d** *SOX2* and *ST6Gal1* copy number in the ovarian cancer lines within the NCI-60 panel

TCGA databases were screened for changes in *SOX2* and *ST6GAL1* copy number. There were 74 cohorts with available data for copy number alterations, spanning a broad range of cancer types. The 74 cohorts were aligned from left to right based on the prevalence of *SOX2* copy number variations (Fig. 1a, upper panel). This same order of cohorts was used to graph the data for *ST6GAL1* (lower panel). For example, the first two bars on the left of the graph represent lung squamous cell carcinoma (SCC) cohorts (tan circles), whereas the third bar, marked with an asterisk (*), depicts an ovarian serous carcinoma cohort (light blue circle). Results in Fig. 1a reveal several notable findings. First, both the *SOX2* and *ST6GAL1* genes are extensively amplified, but rarely deleted, across multiple cancer types. Secondly, in almost every case, *SOX2* and *ST6GAL1* are amplified together. Among the 50 out of 74 cohorts that display copy number alterations in either *SOX2* or *ST6GAL1*, 48 cohorts had amplification of both genes. A third important finding is that there is a striking similarity between the frequency of *SOX2* and *ST6GAL1* copy number gains (CNGs) across the cohorts. For instance, the rates of *SOX2* and *ST6GAL1* CNG within the ovarian serous carcinoma cohort (*) are 27 and 24%, respectively.

The ovarian serous carcinoma cohort (*) was subsequently examined in more detail. This cohort includes specimens from 579 patients. Each individual tumor specimen is represented by a single bar, with red bars indicating patients with CNGs in *SOX2* or *ST6GAL1* (Fig. 1b). As shown, the great majority of patients harboring *SOX2* CNGs also display amplification of *ST6GAL1.* Statistical analyses indicate a significant co-occurrence of CNGs for these two genes (*p* < 0.001). In fact, significant co-occurrence is found for all of the 48 TCGA cohorts with dual *SOX2* and *ST6GAL1* amplification.

Having observed coordinate amplification of *SOX2* and *ST6GAL1* in human tumor tissues, we next examined the copy number status of these genes in the NCI-60 panel of established human cancer cell lines. Again, we noted a strong association between cells with high copy numbers of *SOX2* and *ST6GAL1* (Fig. 1 c). Furthermore, the ovarian cancer cell lines within the NCI-60 panel showed a clear correlation between *SOX2* and *ST6GAL1* CNGs (Fig. 1 d). Taken together, the data in Fig. 1 suggest that *SOX2* is rarely amplified without simultaneous amplification of *ST6GAL1*. While co-amplification doesn’t directly infer a functional relationship between these two genes, these data hint at a selective process whereby amplification of these two genes may be beneficial to the malignancy.

### Levels of ST6Gal-I and Sox2 protein are correlated in ovarian cancer cell lines

**Fig. 2:**
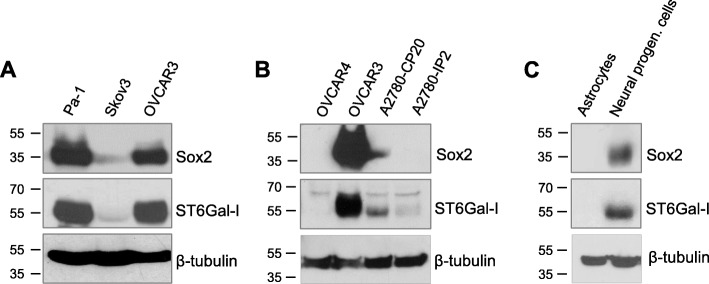
Sox2 and ST6Gal-I expression levels correspond in ovarian cancer cell lines. Immunoblots for ST6Gal-I and Sox2 expression in: **a** ovarian cancer cell lines, Pa-1, Skov-3, and OVCAR3; **b** ovarian cancer cell lines, OVCAR 4, OVCAR3, A2780-CP20 (cisplatin-resistant), and A2780-IP2 (cisplatin-sensitive); and **c** normal human astrocytes and neural progenitor cells

We next evaluated levels of Sox2 and ST6Gal-I protein in multiple ovarian cancer cell lines. Figure 2a depicts immunoblots from the following lines: Pa-1 (derived from an ovarian teratocarcinoma [54]); Skov3 (thought to be a clear cell ovarian cancer line [55, 56]); and OVCAR3 (derived from high-grade serous ovarian adenocarcinoma [57]). Sox2 and ST6Gal-I protein were found to be highly expressed in Pa-1 and OVCAR3 cells, whereas expression of these two proteins was relatively low in Skov3 cells. A similar correspondence in Sox2 and ST6Gal-I protein levels was observed in additional ovarian cancer cell lines (Fig. 2 b). The OVCAR4 line is one of the few ovarian cancer lines that lacks detectable ST6Gal-I protein (unpublished observation), and Sox2 is likewise undetectable in this line. We also compared Sox2 and ST6Gal-I expression in the matched, isogenic chemosensitive/chemoresistant A2780 ovarian cancer cell series, specifically, the A2780-IP2 line, which is sensitive to cisplatin, and the A2780-CP20 line, which is cisplatin-resistant. We were interested in this model because we previously observed enhanced ST6Gal-I expression in cisplatin-resistant A2780-CP20 cells [22]. As shown in Fig. 2b, both Sox2 and ST6Gal-I were upregulated in A2780-CP20 cells compared with A2780-IP2 cells. These data are consistent with the known roles of Sox2 and ST6Gal-I in chemoresistance, a phenotypic hallmark of CSCs [25, 58, 59].

In addition to promoting CSC characteristics, Sox2 and ST6Gal-I have been implicated in the maintenance of stem-like properties in nonmalignant cells [37, 60, 61]. As well, ChIPSeq results point to Sox2 as a regulator of ST6Gal-I expression in stem/progenitor populations [62–64]. To examine the relationship between Sox2 and ST6Gal-I in nonmalignant populations with changes in differentiation status, immunoblotting was conducted on lysates from neural progenitor cells or human astrocytes. As shown in Fig. 2c, levels of Sox2 and ST6Gal-I protein were very high in neural progenitor cells, but undetectable in astrocytes. These data support the concept that, like Sox2, ST6Gal-I expression is enriched in progenitor cells.

### Ovarian cancer cells primarily express the P3-driven ST6Gal-I mRNA isoform

**Fig. 3:**
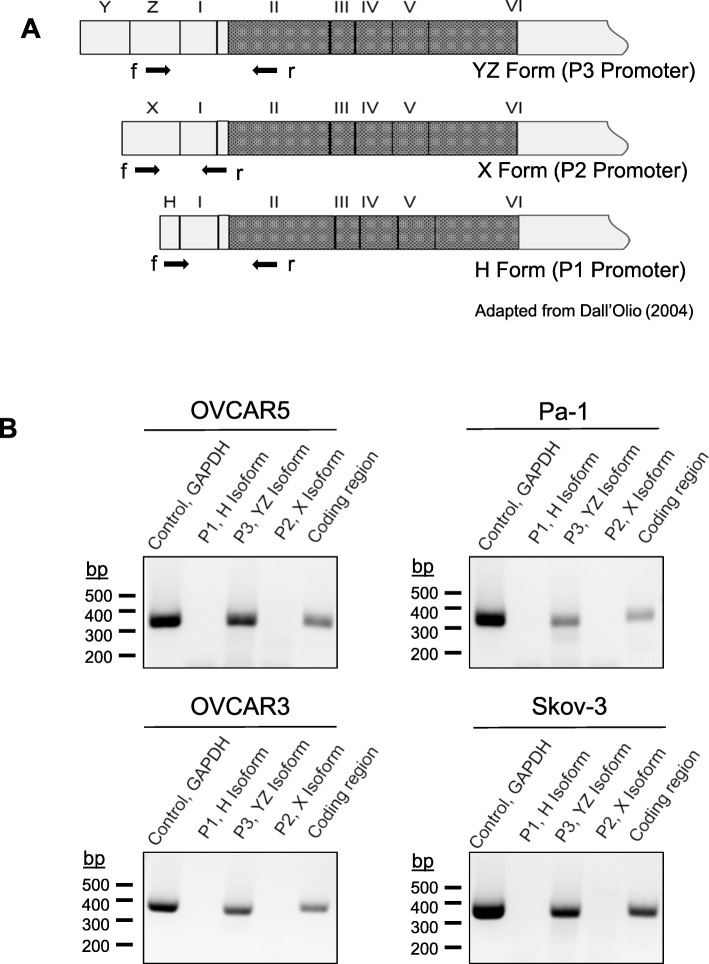
ST6Gal-I expression is driven primarily by the P3 promoter. **a** Graphical representation of the 3 promoter-specific mRNA isoforms of ST6Gal-I (adapted from Dall’Olio et al. [26]). Shaded region indicates the coding sequence, which begins in Exon II and spans through part of Exon VI. **b** ST6Gal-I mRNA isoforms were amplified by PCR using isoform-specific primers. The PCR products were resolved on agarose gels, and detected with ethidium bromide. The expected sizes for the PCR products are 284 bp for the H form, 363 bp for the YZ form, and 253 bp for the X form. We also performed PCR with primers for the ST6Gal-I coding region (expected product = 372 bp), along with primers for GAPDH as a control (expected product = 371 bp)

The strong correlation between Sox2 and ST6Gal-I expression is likely due, at least in part, to the coordinate amplification of these two genes in cancer cells. However, in addition to this mechanism, a ChIP study suggested that Sox2 may regulate ST6Gal-I transcription [38]. Hence, we investigated whether Sox2 binds to the *ST6GAL1* promoter. To address this hypothesis, we first determined which *ST6GAL1* promoter was the primary driver of ST6Gal-I expression in ovarian cancer cells. The schematic diagram in Fig. 3a depicts three of the major ST6Gal-I mRNA isoforms. The “YZ” form, which encompasses exons Y and Z, is driven by the P3 promoter. The “X” form is driven by the P2 promoter, whereas the “H” form is driven by P1. While each isoform contains its own unique transcriptional start site (TSS) and 5′ untranslated region, the coding region, included within exons II-VI, is conserved. Primers were developed for each of the isoforms, as indicated in Fig. 3a, with the 5′ (forward) primer denoted as “f” and the 3′ (reverse) primer denoted as “r.” Expression of the isoforms was assessed in four ovarian cancer cell lines by RT-PCR, followed by gel electrophoresis. As shown in Fig. 3b, the P3-driven YZ variant was the major isoform noted in all of the cell lines (the coding region was also amplified as a positive control).

### Sox2 binds directly to sites proximal to the ST6GAL1 P3 promoter

**Fig. 4:**
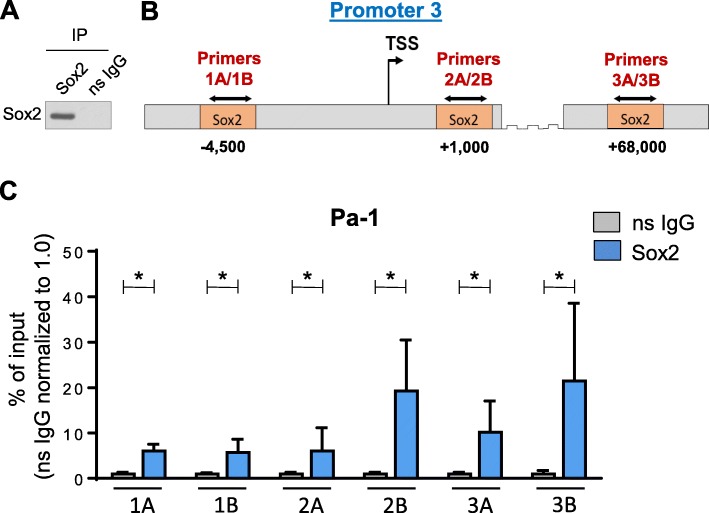
Sox2 binds to multiple regions within the *ST6GAL1* gene. **a** Immunoblot verification of Sox2 immunoprecipitation by the ChIP-validated anti-Sox2 antibody. **b** Schematic depicting three known Sox2-binding elements within the *ST6GAL1* gene (positioned at − 4500, + 1000, and + 68,000 relative to the TSS within the P3 promoter). Primers 1A and 1B were designed to flank the − 4500 site, Primers 2A and 2B flank the + 1000 site, and Primers 3A and 3B flank the + 68,000 site. **c** ChIP assays conducted in Pa-1 ovarian cancer cells show that Sox2 binds all three of the predicted Sox2-binding elements within *ST6GAL1*. * denotes *p* < 0.05

ChIP assays were conducted to determine whether Sox2 binds to the *ST6GAL1* P3 promoter. The Pa-1 cell line was used as a model due to its high expression of Sox2. Prior to performing ChIP assays, we validated the anti-Sox2 ChIP antibody. The anti-Sox2 antibody and a nonspecific IgG control were incubated with chromatin preparations harvested from Pa-1 cells, and the immunoprecipitates were evaluated for Sox2 by immunoblotting. As shown in Fig. 4a, Sox2 was detected in anti-Sox2 immunoprecipitates, but not in control IgG samples. We then utilized the Gene Transcription Regulation Database (GTRD [65]), to identify ChIP-seq-verified Sox2 binding sites on the *ST6GAL1* gene. We focused on three known Sox2 binding elements, a site ~ 4500 bp upstream of the P3 TSS; a site ~ 1000 bp downstream of the TSS, and a site at ~ 68,000 bp downstream of the TSS. While the + 68,000 Sox2 binding element lies within an intron between exons Z and I, recent evidence has underscored an important function for Sox in regulating gene transcription through its binding to distal enhancers [66–68]. To examine Sox2 binding, two distinct sets of primers were designed to flank each of the three binding sites: primers 1A and 1B for the − 4500 bp site; primers 2A and 2B for the + 1000 bp site; and primers 3A and 3B, which bind the + 68,000 bp site (schematic diagram in Fig. 4 b). ChIP experiments were then conducted on Pa-1 cells, and primer binding was quantified by qRT-PCR, with results normalized to input values. As shown in Fig. 4c, significant differences were found between the Sox2 and nonspecific IgG samples for all primer sets, suggesting that Sox2 binds to each of the three ChIP-seq-verified response elements.

### Direct modulation of Sox2 expression alters ST6Gal-I expression and cell surface sialylation

**Fig. 5:**
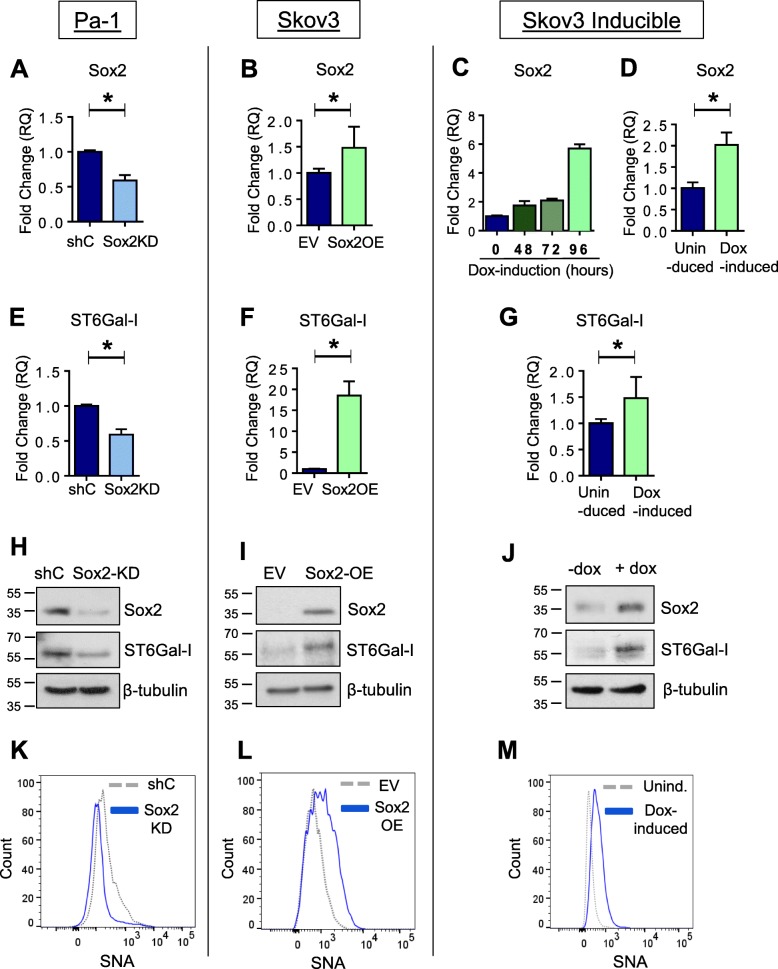
Sox2 promotes expression of ST6Gal-I mRNA and protein, leading to enhanced surface α2–6 sialylation. **a** Sox2 mRNA expression in Pa-1 cells with Sox2 knockdown (KD) or cells transduced with a control shRNA (shC). **b** Skov3 cells with forced overexpression (OE) of Sox2 or cells transduced with a control empty vector (EV). **c** Skov3 cell line with doxycycline (dox)-inducible expression of Sox2. Cells were treated with dox for a time course of 48, 72, or 96 h. **d** Sox2 mRNA expression in cells with inducible Sox2 OE; cells were either induced for 96 h or left uninduced. **e** ST6Gal-I mRNA expression in Pa-1 shC and KD cells. **f** ST6Gal-I mRNA expression in Skov3 EV and OE cells. **g** ST6Gal-I mRNA expression in Skov3 cells with inducible Sox2. Cells were either left uninduced or treated with dox for 96 h. **h-j** Immunoblot for Sox2 and ST6Gal-I in Pa-1 shC and KD cells (**h**), Skov3 EV and OE cells (**i**), or Skov3 inducible cells treated with or without dox treatment for 96 h (**j**). **k-m**. SNA staining to detect α2–6 surface sialylation in Pa-1 shC and KD cells (**k**), Skov-3 EV and OE cells (**l**), and Skov-3 inducible cells with or without dox treatment for 96 h (**m**)**.** * denotes *p* < 0.05

To substantiate a role for Sox2 in regulating ST6Gal-I expression, we modulated Sox2 expression in ovarian cancer cells, and then measured ST6Gal-I mRNA and protein. Sox2 was knocked-down (KD) in the Pa-1 line, given that these cells have high Sox2 expression, and overexpressed (OE) in Skov3 cells, which have relatively low Sox2 expression. Sox2 mRNA levels were evaluated by qRT-PCR to verify successful KD (Fig. 5 a) or OE (Fig. 5 b). We also created a Skov3 line with doxycycline (dox)-inducible Sox2 expression. A pilot time course experiment suggested that a 96 h dox treatment was optimal for inducing Sox2 expression (Fig. 5 c). Further experiments using the 96 h time point confirmed that dox treatment significantly increased Sox2 levels (Fig. 5 d).

Cells were then evaluated for ST6Gal-I mRNA expression. Sox2 KD in Pa-1 cells decreased the expression of ST6Gal-I mRNA (Fig. 5 e), while both constitutive (Fig. 5 f), and inducible (Fig. 5 g), Sox2 OE in Skov3 cells upregulated ST6Gal-I mRNA. These data were validated by immunoblotting experiments, which showed that Sox2 KD inhibits, while Sox2 OE enhances, expression of ST6Gal-I protein (Fig. 5 h-j). Finally, the surface α2–6 sialylation of cells with modulated Sox2 expression was examined as a measure of ST6Gal-I activity. To this end, cells were stained with SNA, a lectin specific for α2–6 sialic acids, and analyzed by flow cytometry. In Pa-1 cells with Sox2 KD, we observed decreased surface α2–6 sialylation (Fig. 5 k) whereas α2–6 sialylation was enriched in both the constitutive and inducible Sox2 OE models (Fig. L-M). In the aggregate, these data suggest that Sox2 directly promotes the expression of ST6Gal-I in ovarian cancer cells, leading to enhanced surface α2–6 sialylation.

## Discussion

A plethora of literature has established Sox2 as a master regulator of stemness. Sox2 plays a seminal part in balancing the transcription of genes that regulate a cell’s differentiation state [43, 60, 69, 70]. Following the discovery that genetic deletion of Sox2 is embryonic lethal, research has focused on the importance of Sox2 in cell fate determination and stem cell maintenance [61, 71, 72]. However, another crucial function for Sox2 has recently emerged: its role in cancer development and promotion of CSCs characteristics [45, 46, 73]. Sox2 activity propels tumor cell migration and invasion [74], self-renewal [45], tumor initiating capacity [47], and chemoresistance [70]. Interestingly, these same CSC features are imparted by ST6Gal-I [9, 12, 16, 22, 25]. In addition to shared functionality, there is evidence suggesting that Sox2 and ST6Gal-I mRNA levels are correlated [38–40], and that ST6Gal-I may, in fact, act as a downstream effector of Sox2 to promote stemness. As an example, Wang et al. showed that knock-down of ST6Gal-I suppressed the transition to pluripotency induced by the four Yamanaka factors, one of which is Sox2 [37]. These findings implicate an interplay between Sox2 and ST6Gal-I that may be crucial for maintaining the cellular properties necessary to support a progenitor-like state.

While an association between Sox2 and ST6Gal-I mRNA levels has been noted previously [38–40], results herein show that Sox2 and ST6Gal-I expression at the protein level is correlated across multiple ovarian cancer cell lines. One potential reason for this correlative expression is that the *SOX2* and *ST6GAL1* genes lie within the same amplicon, 3q26, and are coordinately amplified in the vast majority of tumor specimens. Likewise, there is a strong correspondence between *SOX2* and *ST6GAL1* CNGs in the NCI-60 panel of established cancer cell lines. The 3q26 amplicon is one of the most pervasively amplified chromosomal regions in cancer, and is known for promoting stem-like cancer cell characteristics [48]. This particular amplicon is estimated to occur in greater than 20% of all human cancers [48], with amplification rates as high as 75% in invasive lung SCC [75], and between 25 and 30% in ovarian cancer [51, 76, 77]. In addition to lung SCC and ovarian cancer, 3q26 amplification has been documented in esophageal SCC, head and neck SCC, and cervical cancer [49, 50, 78–80]. The most widely studied genes within 3q26 include *SOX2*, *PRKCI*, *ECT2*, and *PIK3CA*, and these genes work together to promote carcinogenesis [80]. Of note, 3q26 amplification is an early event in tumorigenesis, which aligns with evidence suggesting that Sox2 functions as an initiating oncogene in ovarian cancer [42, 80, 81]. However, while extensive research has focused on *SOX2*, the presence of *ST6GAL1* within the 3q26 amplicon has escaped attention. We postulate that some of the tumor-promoting activity of the 3q26 amplicon may be mediated by *ST6GAL1*.

In conjunction with genetic co-amplification, Sox2 may directly regulate the expression of ST6Gal-I. Our analyses of publically available ChIPSeq databases uncovered two Sox2 binding elements proximal to the *ST6GAL1* P3 promoter, one at ~ 4500 bp upstream, and another ~ 1000 downstream, of the TSS. A third site was identified at ~ 68,000 bp downstream of the P3 TSS, an area that lies within an intron between exons Z and I. Using ChIP assays, we confirmed Sox2 binding to all three of these sites in ovarian cancer cells. These data are noteworthy because prior ChIPSeq experiments were conducted primarily in stem cell models, or other types of cancers. More specifically, ChIPSeq databases show that Sox2 binds to the ~ 4500 bp site upstream of the TSS in ESC-derived neural progenitor cells (NPCs) [62], whereas the + 1000 bp site is bound by Sox2 in NPCs, ESCs, and iPSCs [62–64, 82]. Sox2 similarly associates with the 1000+ bp site in select cancer cell populations such as thyroid gland medullary carcinoma and esophageal SCC [64] The third site at ~ 68,000 bp downstream of the TSS is bound by Sox2 in ESCs and iPSCs [62, 63, 82–84]. Intriguingly, this latter site is in close proximity to Oct4 and Nanog binding sites [82, 84]. These findings, combined with our own, suggest that Sox2 regulates ST6Gal-I expression in a variety of stem and cancer cell populations.

We further established that Sox2 promotes ST6Gal-I expression by modulating Sox2 levels and examining ST6Gal-I mRNA and protein. While Sox2-induced ST6Gal-I protein expression has not been previously reported, other groups have described Sox2-mediated changes in ST6Gal-I mRNA. In a glioblastoma (GBM) model focused on the regulation of cell plasticity, Berezovsky et al. found that knock-down of Sox2 resulted in a 3.9 fold decrease in ST6Gal-I mRNA; this study also revealed that Sox2 was essential for the maintenance of GBM CSCs [40]. Zhu et al. investigated Sox2 as a marker for stem-like bladder cancer cells and determined that overexpression of Sox2 induced an increase in ST6Gal-I mRNA [39]. A third group evaluated Sox2 in the context of SCC of the skin [38]. Sox2 knock-in mice with SCC had elevated mRNA levels of ST6Gal-I whereas ST6Gal-I mRNA was reduced in Sox2 knockout mice [38]. While these studies support a correlation between Sox2 and ST6Gal-I, our study provides an advance by showing that Sox2 activity leads to enhanced protein expression and importantly, increased cell surface α2–6 sialylation. It is well known that the phenotypic effects of ST6Gal-I are directed by enriched receptor sialylation, which correspondingly modulates intracellular signaling networks [11–13].

The dearth of information regarding mechanisms that mediate differential ST6Gal-I expression in diverse cell types represents a major gap in the field. In particular, very little is known about ST6Gal-I regulation in stem and progenitor cells. On the other hand, there is some insight into pathways that induce ST6Gal-I expression in cancer cells. Several groups, including ours, have shown that ST6Gal-I is upregulated in response to signaling by oncogenic ras [27, 85, 86]. Piller’s group further determined that activated ras acts through RalGEF to promote expression of the P3-driven ST6Gal-I isoform [27]. ST6Gal-I has also been identified as part of a BRAF-driven metastatic gene signature in melanoma [87]. Moreover, ST6Gal-I is upregulated via SP-1-dependent transcription during TGFβ-induced epithelial to mesenchymal transition in breast cancer cells [88]. Finally, cytokines typically present within the tumor microenvironment, such as TNF, IL-1, and IL-6, have been shown to increase ST6Gal-I expression [89, 90]. The current study adds to the body of literature by highlighting a novel role for Sox2 in promoting expression of ST6Gal-I in ovarian cancer cells.

## Conclusions

Studies herein describe a complex relationship between Sox2 and ST6Gal-I. Both genes are commonly amplified in cancer cells due to their shared presence within the 3q26 amplicon, and then the transcribed Sox2 protein binds the *ST6GAL1* promoter to further enhance ST6Gal-I expression. These collective events may contribute to the remodeling of cancer cells into a more stem-like cell phenotype.

## Data Availability

All data generated and analyzed during the course of this study are either included in this manuscript, or available through The Cancer Genome Atlas website (https://www.cancer.gov/about-nci/organization/ccg/research/structural-genomics/tcga).
